# Prevalence of Post-Traumatic Stress Disorder in Emergency Physicians in the United States

**DOI:** 10.5811/westjem.2019.7.42671

**Published:** 2019-08-28

**Authors:** Joseph A. DeLucia, Cindy Bitter, Jennifer Fitzgerald, Miggie Greenberg, Preeti Dalwari, Paula Buchanan

**Affiliations:** * Saint Louis University School of Medicine, Department of Surgery/Division of Emergency Medicine, Saint Louis, Missouri; † Saint Louis University School of Medicine, Department of Psychiatry, Saint Louis, Missouri; ‡ Saint Louis University, Saint Louis University Center for Health Outcomes Research, Saint Louis, Missouri

## Abstract

**Introduction:**

There is increasing concern about the effects of occupational stressors on the wellness of healthcare providers. Given high patient acuity, circadian rhythm disruption, and other workplace stressors, emergency physicians (EP) would be predicted to have high rates of occupational stress. We conducted this study to assess the prevalence of post-traumatic stress disorder (PTSD) in attending EPs practicing in the United States.

**Methods:**

A link to an electronic questionnaire was distributed through the emergency medicine-centric publication *Emergency Medicine News*. We compared the prevalence of PTSD in EPs to the general population using a chi-square goodness of fit test, and performed logistic regression to assess for significance of risk factors.

**Results:**

We received survey responses from 526 persons. In this study, EPs had a PTSD point prevalence of 15.8%. Being a victim of a prior trauma or abuse is the primary predictor of PTSD (odds ratio [OR] [95% confidence interval {CI}, 2.16 (1.21 – 3.86)], p = 0.009) and PTSD severity score (OR [95% CI, 1.16 (1.07 – 1.26)], p <0.001).

**Conclusion:**

Emergency physicians have a substantial burden of PTSD, potentially jeopardizing their own health and career longevity. Future studies should focus on identifying subgroups at higher risk for PTSD and modifiable risk factors. Prevention and treatment strategies should be developed and tested in healthcare providers.

## INTRODUCTION

Post-traumatic stress disorder (PTSD) affects some people who have been exposed to traumatic events such as military action, natural disasters, sexual violence, or serious illness/injury. In the United States (U.S.), the point prevalence of PTSD in adults is estimated to be 3.8%. The diagnosis of PTSD requires an exposure to trauma and symptoms from multiple domains, including intrusive memories, avoidance, negative mood, and hyperarousal. Symptoms must occur for more than one month and cause functional impairment to meet criteria for PTSD. Exposure was originally defined as personal experience, witnessing events, or indirect exposure through events that occurrred to loved ones.

The terms secondary traumatic stress (STS) and compassion fatigue were used to describe the emotional toll suffered by persons who have repeated but indirect exposure to trauma as part of their professional or volunteer duties, such as healthcare workers, firefighters, forensic examiners, and humanitarian workers. In recognition of a growing body of literature suggesting STS has a profound effect on workers in these fields, the 2013 update to the Diagnostic and Statistical Manual of Mental Disorders (fifth edition) (DSM-5) added repeated indirect exposure as an exposure class.

Physicians have high rates of substance abuse and suicide, which may be mediated by underlying PTSD.^6^–^8^ STS and PTSD have been described in many types of healthcare providers, but emergency physicians (EP) may be particularly vulnerable. EPs deal with multiple challenges such as the potential to witness death and trauma on a frequent basis, diagnostic uncertainty, high patient acuity, crowding, and circadian rhythm disruption that place them as elevated risk for occupational stress. A single-site study from the U.S. found that 11.9% of emergency medicine (EM) residents met criteria for PTSD, with 30% having symptoms that did not meet the threshold for diagnosis.^9^ A study of EPs and advance practice providers from a group practice in the U.S. found a PTSD prevalence of 12.7%.^10^ Research from other countries corroborates this vulnerability, with prevalence of self-assessed PTSD of 16.8% in German EPs, 15.4% in Pakistani EPs, and 14.5% in Belgian EPs.^11^–^13^

The objective of this study was to determine the point prevalence of PTSD in a cohort of practicing EPs from multiple practice settings in the U.S., and to compare this to the prevalence in the general population. The secondary objective was to determine if personal or practice-related factors mediate prevalence of PTSD. Determining the prevalence of PTSD in EPs and identifying high-risk subgroups will hopefully improve methods to prevent and treat PTSD in EPs and other healthcare providers.

## METHODS

### Study Design

We developed a questionnaire using a validated PTSD screening tool (Appendix 1) and demographic factors predicted to mediate risk, based on a review of the literature.14 A short background article with a recruitment statement and a link to the electronic survey was distributed through *Emergency Medicine News*. The survey was advertised once in December 2015 and was open for completion through April 2016. Completion of the survey was entirely voluntary and anonymous. The online survey company (Qualtrics, Provo, Utah) collected the data and forwarded results to the research team. The study protocol was approved by the Institutional Review Board at Saint Louis University.

### Selection of Participants

Participants were voluntary and self-selected from the readership of *Emergency Medicine News*. The newsletter is distributed free of charge to practicing (postgraduate) EPs. The recruitment statement specified that respondents should be practicing EPs; there were no exclusion criteria listed. We attempted to prevent duplication of participants by electronically collecting participant data and computer internet protocol numbers. Once this data was recorded, the survey would not re-open for the same data and computer internet protocol numbers.

**Population Health Research Capsule**

What do we already know about this issue?

*Emergency physicians have multiple occupational stressors that may increase their risk for post-traumatic stress disorder (PTSD). Previous single site studies have found a prevalence of 12–13% in emergency medicine practitioners*.

What was the research question?

*Using a validated screening tool, the study aimed to determine the point-prevalence of PTSD in emergency physicians from the United States who practice in a variety of settings*.

What was the major finding of the study?

*The point prevalence of PTSD in emergency physicians is 15.8%. A history of prior exposure to trauma was the only independent risk factor for meeting the screening threshold for PTSD*.

How does this improve population health?

*Understanding the magnitude of the problem and risk factors for development of PTSD will hopefully drive development of interventions to reduce occupational stress in emergency physicians*.

### Methods and Measurements

The PTSD screening tool, PTSD Checklist – Civilian Version (PCL-C) (Appendix 1), was entered into an online survey tool. It consists of 17 questions that are answered on a five-point Likert scale, based off of experiences in the prior month. From this survey we calculated a total PTSD severity score (range = 17–85) and defined PTSD according to the DSM criteria, which was a symptomatic response to at least 1 “B” item, at least 3 “C” items, and at least 2 “D” items. Symptomatic responses were defined as those in the categories of “Moderately” or above (3 on Likert scale of 1–5).^1^ The PCL-C has been validated as a screening tool as well as an adjunct to the clinical interview for the diagnosis of PTSD, with an estimated sensitivity of 0.70, specificity of 0.90, and a positive likelihood ratio of 6.8.^15^–^16^

Additional covariates we collected included gender, age (22 – 28, 29 – 35, 36 – 42, 43 – 49, 50 – 56, 57 – 63, 63 – 70, > 70 years); board certification (EM, family practice, internal medicine and pediatrics); years of service (0 – 5, 6 – 11, 12 – 17, 18 – 23, 24 – 29, > 29 years); location of work (urban, suburban, or rural); trauma level status (I, II, III, IV, or “None”); military experience (yes/no); marital status (single, married or domestic partner); whether they had children (yes/no); and whether they were a prior victim of trauma or abuse (yes/no).

### Statistical Analysis

We used descriptive statistics to analyze participants’ demographic characteristics, their overall PTSD severity score, and whether or not they met criteria for diagnosis of PTSD. Chi-square goodness of fit test was used to determine whether the prevalence of PTSD in EPs was similar to that of the general population.

Independent samples t-tests, one-way analysis of variance (ANOVA) tests and Spearman correlations were used to assess the association between dichotomous, multi-categorical, and ordinal patient characteristics, respectively, with total PSTD score. In the case of a significant ANOVA test, we applied Bonferroni corrections to adjust for multiple significance testing in the post hoc tests. We assessed bivariate analysis of the ordinal characteristics, age, years of service, and trauma level with whether or not the patient had PTSD using Kruskal-Wallis tests, while categorical characteristics used the chi-square test. We conducted a multiple linear regression on the outcome of the natural log of PTSD score to assess potential predictors of score and a multiple logistic regression on whether or not the subject had PTSD. For both outcomes we conducted bivariate regressions on each characteristic, and if the characteristic had a p value <0.20, we then included it in the multivariate regression model. Due to low counts, categories of some characteristics were combined to increase the count. We performed analyses using SPSS Statistics for Windows version 23 (IBM Corp., Armonk, NY). Statistical tests were two-tailed and the significance level set at p < 0.05.

## RESULTS

Table 1 shows the demographic characteristics of the study participants. There were 526 participants in the study with 56.1% males, 82.3% married, and approximately 50% having less than 11 years of service. Those who reported being a victim of trauma or abuse made up 15.8% of the participants. The majority of respondents worked in suburban or urban locations.

The breakdown of the components of the PTSD severity score as well as the total PTSD severity score are listed in Appendix 2. Difficulty falling or staying asleep was the most common criterion of PTSD that the subjects reported being bothered by during the prior month, with 37 (7%) reporting being extremely bothered. The mean total PTSD score was 31.1 (standard deviation [SD] =11.7). Of the total sample, 83 (15.8%) met criteria for a diagnosis of PTSD according to the DSM-5. This is significantly higher than the prevalence of PTSD in the general population (point prevalence 3.8%, p<0.001).

The relationship between demographic covariates, total PTSD severity score, and meeting criteria for PTSD are shown in Table 2. The mean (SD) PTSD score for those who reported being a victim of trauma or abuse was 35.9 (14.0), statistically different than that of non-victims 30.2 (11.0), p = 0.001. Bivariate analysis showed no statistically significant difference between mean PTSD severity score by age, gender, marital status, having children, or military service. Those who met the DSM criteria for PTSD tended to be older, median (interquartile range [IQR]) PTSD: 43–49 years (43–49 years – 50–56 years) vs. no PTSD: 36 – 42 years (26–42 years – 50–56 years), p =0.001. Subjects who met criteria for PTSD were also more likely to have served in the military (20.5% vs 11.1%, p = 0.017). Subjects with prior trauma had a higher risk for meeting the criteria for PTSD than those without prior trauma (28.9% vs 13.3%, p<0.001).

The influence of workplace variables, total PTSD severity score, and meeting criteria for PTSD is shown in Table 3. We found no significant correlation between years of service, or being board certified in EM, family medicine or internal medicine, and the PTSD severity score. Those who were board certified in pediatric medicine had a lower mean (SD) PTSD severity score than those not certified 24.7 (7.8) vs 31.3 (11.8), (p = 0.04). In addition, there was a weak but significant correlation between facility trauma level and PTSD severity score (r_s_ = 0.12, p = 0.006). The median (IQR) of years of service was 12–17 years (6–11 years – 24–29 years) for those with PTSD and 6–11 years (6–11 years – 18–23 years) for those without PTSD, p = 0.003.

Appendix 3 shows relationships between predictor variables and PTSD severity score in the multivariate model. After adjusting for age, marital status, military service, being a victim of a past trauma, trauma level at practice site, location of work, being board certified in EM, and being board certified in pediatric medicine, only being a victim of a past trauma and hospital trauma level were significant predictors of PTSD severity score. Prior victims had a 16% increase in PTSD severity score (95% confidence interval [CI], 7–26%, p <0.001), and those working at a trauma level II hospital had a 10% increase in PTSD severity score (95% CI, 1–20%, p = 0.03) compared to those working at a level I trauma center or those working at trauma level III/IV hospitals.

Predictors of meeting criteria for PTSD can be found in Appendix 4. After adjusting for age, gender, marital status, prior military service, being a victim of a previous trauma, years of service, and being board certified in EM, only being a victim of a previous trauma was a significant predictor of PTSD. Those who were a victim were more than twice as likely to be diagnosed with PTSD as those who were not a victim (odds ratio OR = 2.16, 95% CI, 1.21 – 3.86, p = 0.009).

## DISCUSSION

In this study, the point prevalence of self-assessed PTSD in EPs was 15.8%. PTSD severity scores were higher among victims of prior trauma and physicians working at trauma level II hospitals. Greater age, prior military service, increased years of service, and a history of prior victimization were associated with meeting the criteria for PTSD. However, being a victim of prior trauma was the only significant risk factor for PTSD in the multivariate model.

Prevalence of PTSD among resident physicians in the U.S. ranges from 5.2% in medicine and pediatrics, to 22% in surgical residents and 29% in EM residents.^17^–^20^ Intensivists, who deal with many of the same occupational stressors as EPs, have a reported PTSD prevalence of 13%.^21^ In one study, surgeons had a PTSD prevalence of 15% while trauma surgeons had a prevalence of 17%, not significantly different.^22^ A survey of trauma surgeons using the PCL-C found symptoms of PTSD in 40%; 15% met criteria for PTSD. In that study, PTSD symptoms were higher in male surgeons, surgeons who had more operative cases, surgeons who had more than seven call shifts per month, and those who designated less time for relaxation. Development of PTSD was higher in surgeons managing more than five critical cases per call.^23^

PTSD is associated with professional quality of life, burnout, intent to change careers, risk of occupational injury, and markers of provider health such as sleep quality and obesity.^24^–^26^ There is increasing evidence that burnout and PTSD among providers worsen patient outcomes.^27^–^28^

Not all persons who suffer a trauma develop symptoms of PTSD. Risk factors for development of PTSD in the general population include underlying psychiatric problems, concurrent medical illness, history of being a victim of child abuse, higher degree of acute stress symptoms, and more severe trauma as the inciting event.^29^–^31^ Psychological traits of neuroticism and dissociation, use of maladaptive coping strategies such as disengagement, and cognitive factors such as self-perceived resilience and suppression of emotion predicted a higher risk of developing PTSD in newly trained paramedics.^32^ Potential healthcare-specific risk factors for PTSD include workplace violence, bullying, the death of a child, fear of exposure to infectious disease, litigation stress, use of electronic health records, long work hours, and circadian disturbance due to night shifts.^18^, ^27^–^28^, ^33^–^35^ Bellolio et al. found that working primarily night shifts and working more than 80 hours per week were predictive of burnout, but found no increased risk based on specialty when these factors were controlled.^36^

One surprising finding in this study was that physicians who practiced in level II trauma centers had a higher PTSD scores than physicians working in either level I centers or level III/IV centers. It might be predicted that physicians working in facilities with a lower burden of severe trauma would have a lower prevalence of PTSD; thus, the mechanism for higher PTSD scores in physicians at level II centers is unclear. We postulate that shared responsibility with in-house trauma surgeons and the availability of other resources not found at level II hospitals may be protective for physicians at level I hospitals. Total hours worked per month and other scheduling factors may also vary between level I and II hospitals. To our knowledge, this has not been reported previously.

There are also protective factors that may prevent development of PTSD. Having good family and workplace support are the most important, but use of light-hearted humor and adaptive coping strategies are also protective.^37^–^42^ A strong professional identity is important for resilience; however, one study suggested a higher sense of calling may be hazardous to practitioners exhibiting early signs of PTSD.^43^–^44^

Current research suggests that job burnout precedes development of PTSD; thus, interventions to increase resilience and reduce burnout should ameliorate the prevalence of PTSD in healthcare workers.^45^ Research in rescue workers suggests that early symptoms of emotional distress predict long-term sequelae.^46^ Most resilience interventions in healthcare workers are based on mindfulness training or cognitive behavioral therapy.^47^ Studies of resilience training in EM have shown mixed results.^48^–^49^ While personal resilience is important to prevent burnout and PTSD, a recent systematic review and meta-analysis of programs to reduce burnout in physicians suggests that focusing on adaptations to the work environment are more effective than interventions that target individual providers.^50^ Noben et al found that an intervention to improve mental health among hospital staff was cost-effective.^51^

## LIMITATIONS

Limitations include the small number of respondents to the survey. *Emergency Medicine News* reported a readership of 38,909 during that time frame (personal communication, Wolters Kluwer). People are more likely to respond to surveys if the topic is of personal interest, eg, because they are affected by the items asked about. People who respond almost certainly have different characteristics than those who do not, causing selection bias.^51^ However, the prevalence of self-assessed PTSD in our study was similar to the prevalence reported in multiple other countries. To the best of our knowledge, this study is the largest multisite study to assess the prevalence of PTSD in EPs in the United States.

Although the EM news magazine is primarily distributed to practicing physicians, it is possible that residents, medical students, or other healthcare providers may have seen the survey link and responded. The term “prior victim of trauma or abuse” was not specifically defined, which may have resulted in some mis-categorization. Additionally, this survey could not determine the contribution of work-related stressors to the baseline prevalence of PTSD in EPs. Like many highly stressed professionals who are under constant scrutiny, EPs may not report symptoms of PTSD. Having PTSD may be perceived as a weakness or inability to do one’s job. There may be fears that once diagnosed, hours may be cut back or one may be re-assigned to less-stressful work areas. This can lead to loss of self-confidence and respect.

## CONCLUSION

There is a substantial burden of PTSD among practicing emergency physicians. Additional large-scale studies should be done to more accurately assess the prevalence of PTSD symptoms in EPs, modifiable risk factors for development of PTSD, the relationship between PTSD, burnout, and career longevity, and the effects of interventions currently underway within the specialty. Interventions at the organizational level should be prioritized.

## Supplementary Information









## Figures and Tables

**Table 1:** Characteristics of physician participants, n = 526, in study examining prevalence of post-traumatic stress disorder.

Demographic Factors	n (%)
Age groups (years)	
22–28	5 (1.0)
29–35	109 (20.7)
36–42	146 (27.8)
43–49	95 (18.1)
50–56	85 (16.2)
57–63	61 (11.6)
63–69	23 (4.4)
70+	2 (0.4)
Gender	
Male	295 (56.1)
Female	230 (43.7)
Unknown	1 (0.2)
Board EM	
Yes	488 (92.8)
No	38 (7.2)
Board Family Medicine	
Yes	17 (3.2)
No	509 (96.8)
Board Internal Medicine	
Yes	23 (4.4)
No	503 (95.6)
Board Pediatrics	
Yes	14 (2.7)
No	512 (97.3)
Years of service	
0–5	118 (22.4)
6–11	149 (28.3)
12–17	89 (16.9)
18–23	67 (12.7)
24–29	57 (10.8)
30+	46 (8.7)
Location of work	
Urban	221 (42.0)
Suburban	243 (46.2)
Rural	62 (11.8)
Trauma	
One	141 (26.8)
Two	150 (28.5)
Three	92 (17.5)
Four	23 (4.4)
None	120 (22.8)
Military	
Yes	66 (12.5)
No	460 (87.5)
Marital status	
Married	433 (82.5)
Domestic partner	15 (2.9)
Single	78 (14.8)
Children	
Yes	419 (79.7)
No	107 (20.3)
Victim	
Yes	83 (15.8)
No	443 (84.2)

*EM*, emergency medicine.

## References

[1] Larsen SE, Pacella ML (2016). Comparing the effect of DSM-congruent traumas vs. DSM-incongruent stressors on PTSD symptoms; a meta-analytic review. J Anxiety Disord.

[2] Kilpatrick D, Resnick H, Milanak M (2014). National estimates of exposure to traumatic events and PTSD prevalence using DSM-IV and DSM-5 criteria. J Trauma Stress.

[3] American Psychiatric Association (1980). Diagnostic and Statistical Manual of Mental Disorders.

[4] Figley CR (1995). Compassion fatigue: coping with secondary traumatic stress disorder in those who treat the traumatized. Brunner/Mazel psychological stress series 23.

[5] American Psychiatric Association (2013). Diagnostic and Statistical Manual of Mental Disorders.

[6] Cottler L, Ajinkya S, Merlo L (2013). Lifetime psychiatric and substance use disorders among impaired physicians in a physicians health program: cComparison to a general treatment population. J Addict Med.

[7] Schernhammer E, Colditz G (2004). Suicide rates among physicians: a quantitative and gender assessment (meta-analysis). Am J Psychiatry.

[8] Stewart SH, Pihl RO, Conrod PJ, Dongier M (1998). Functional associations among trauma, PTSD, and substance-related disorders. Addict Behav.

[9] Mills L, Mills T (2005). Symptoms of post-traumatic stress disorder among emergency medicine residents. J Emerg Med.

[10] Roden-Foreman J, Bennett M, Rainey E (2017). Secondary traumatic stress in emergency medicine clinicians. Cogn Behav Ther.

[11] Pajoknk F, Cransac P, Teichmann A (2012). Trauma and stress-related disorders in German emergency physicians: the predictive role of personality factors. Int J Emerg Mental Health.

[12] Zafar W, Khan UR, Siddiqui SA (2016). Workplace violence and self-reported psychological health: coping with post-traumatic stress, mental distress, and burnout among physicians working in the emergency departments compared to other specialties in Pakistan. J Emerg Med.

[13] Somville F, DeGucht V, Maes S (2016). The impact of occupational hazards and traumatic events among Belgian emergency physicians. Int J Behav Nutr Phys Act.

[14] Weathers FW, Litz BT, Keane TM (2013). The PTSD Checklist for *DSM-5* (PCL-5).

[15] Conybeare D, Behar E, Solomon A (2012). The PTSD Checklist-Civilian Version: reliability, validity and factor structure in a nonclinical sample. J Clin Psych.

[16] Spoont MR, Williams JW, Kehle-Forbes S (2015). Does this patient have posttraumatic stress disorder?. JAMA.

[17] Kannan L, Wheeler DS, Blumhof S (2019). Work related post traumatic stress disorder in medicine residents. Acad Psychiatry.

[18] Hollingswort CE, Wesley C, Huckridge J, Finn GM, Griksaitis MJ (2018). Impact of child death on paediatric trainees. Arch Dis Child.

[19] Jackson T, Provencio A, Bentley-Kumar K (2017). PTSD and surgical residents: everybody hurts… sometimes. Am J Surg.

[20] Luftman K, Aydelotte J, Rix K (2017). PTSD in those who care for the injured. Injury.

[21] Colville GA, Smith JG, Brierley J (2017). Coping with staff burnout and work-related posttraumatic stress disorder in intensive care. Pediatr Crit Care Med.

[22] Jackson TN, Morgan JP, Jackson DL (2019). The crossroads of posttraumatic stress disorder and physician burnout: a national review of United States trauma and nontrauma surgeons. Am Surg.

[23] Joseph B, Pandit V, Hadeed G (2014). Unveiling posttraumatic stress disorder in trauma surgeons: a national survey. J Trauma Acute Care Surg.

[24] Laposa J, Alden L, Fullerton L (2003). Work stress and posttraumatic stress disorder in ED nurses/personnel. Emerg Nurs.

[25] Wild J, Smith K, Thompson E (2016). A prospective study of pre-trauma risk factors for post-traumatic stress disorder and depression. Psychol Med.

[26] Katsavouni F, Bebetsos E, Malliou P (2016). The relationship between burnout, PTSD symptoms and injuries in firefighters. Occup Med.

[27] Ruitenburg M, Frings-Dresen M, Sluiter J (2012). The prevalence of common mental disorders among hospital physicians and their association with self-reported work ability: a cross-sectional study. BMC Health Serv Res.

[28] Sendler J, Rutkowska A, Makara-Studzinska M (2016). How the exposure to trauma has hindered physicians’ capacity to heal: prevalence of PTSD among healthcare workers. Eur J Psychiatry.

[29] Brewin C, Andrews B, Valentine J (2000). Meta-analysis of risk factors for posttraumatic stress disorder in trauma-exposed adults. J Consult Clin Psychol.

[30] Haagsma J, Ringburg A, van Lieshout E (2012). Prevalence rate, predictors and long-term course of probable posttraumatic stress disorder after major trauma: a prospective cohort study. BMC Psych.

[31] Chen Y, Wang F, Wen J (2014). Risk factors for post-traumatic stress disorder (PTSD) after Wenchuan earthquake: a case control study. PLoS ONE.

[32] Wild J, Smith K, Thompson E (2016). A prospective study of pre-trauma risk factor for post-traumatic stress disorder and depression. Psychol Med.

[33] Lanctot N, Guay S (2014). The aftermath of workplace violence among healthcare workers: a systematic literature review of the consequences. Aggress Violent Behav.

[34] Shi L, Wang L, Jia X (2017). Prevalence and correlates of symptoms of post-traumatic stress disorder among Chinese healthcare workers exposed to physical violence: a cross-sectional study. BMJ Open.

[35] Stehman CR, Testo Z, Gershaw RS, Kellogg AR (2019). Burnout, drop out, suicide: physician loss in emergency medicine, part 1. West J Emerg Med.

[36] Bellolio MF, Cabrera D, Sadosty AT (2014). Compassion fatigue is similar in emergency medicine residents compared to other medical and surgical specialties. West J Emerg Med.

[37] Haber Y, Palgi Y, Hamama-Raz Y (2013). Predictors of professional quality of life among physicians in a conflict setting: the role of risk and protective factors. Isr J Psychiatry Relat Sci.

[38] Ager A, Pasha E, Yu G (2012). Stress, mental health, and burnout in national humanitarian aid workers in Gulu, Northern Uganda. J Trauma Stress.

[39] Craun S, Bourke M (2014). The use of humor to cope with secondary traumatic stress. J Child Sex Abuse.

[40] McGarry S, Girdler S, McDonald A (2013). Paediatric health-care professionals: relationships between psychological distress, resilience and coping skills. J Paediatr Child Health.

[41] Hamid A, Musa S (2017). The mediating effects of coping strategies on the relationship between secondary traumatic stress and burnout in professional caregivers in the UAE. J Ment Health.

[42] Colville G, Smith J, Brierley J (2017). Coping with staff burnout and work-related posttraumatic stress in intensive care. Pediatric Crit Care Med.

[43] Winkel AF, Robinson A, Jones AA, Squires AP (2019). Physician resilience: a grounded theory study of obstetrics and gynaecology residents. Med Educ.

[44] Jo I, Lee S, Sung G (2018). Relationship between burnout and PTSD symptoms in firefighters: the moderating effects of a sense of calling to firefighting. Int Arch Occup Environ Health.

[45] Shoji K, Lesnierowska M, Smoktuowicz E (2015). What comes first, job burnout or secondary traumatic stress? Findings from two longitudinal studies from the U.S. and Poland. PLoS One.

[46] Kawashima Y, Nishi D, Noguchi H (2016). Post-traumatic stress symptoms and burnout among medical rescue workers 4 years after the Great East Japan Earthquake: a longitudinal study. Disaster Med Public Health Prep.

[47] Cleary M, Kornhaber R, Thapa DK, West S, Visentin D (2018). The effectiveness of interventions to improve resilience among health professionals: a systematic review. Nurse Educ Today.

[48] Dunn PJ, Lynch J, Prihodova L (2019). Burnout in the emergency department: randomized controlled trial of an attention-based training program. J Intregr Med.

[49] Hart D, Paetow G, Zarzar R (2019). Does implementation of a corporate wellness initiative improve burnout?. West J Emerg Med.

[50] Panagioti M, Panagopoulou E, Bower P (2017). Controlled interventions to reduce burnout in physicians: a systematic review and meta-analysis. JAMA Int Med.

[51] Noben C, Evers S, Nieuwenhuijsen K (2015). Protecting and promoting mental health of nurses in the hospital setting: Is it cost-effective from a employer’s perspective?. Int J Occup Med Environ Health.

