# Supplementary material for: Prevalence of Post-Traumatic Stress Disorder in Emergency Physicians in the United States
Source: West J Emerg Med. 2019 Aug 28;20(5):740–6. doi: 10.5811/westjem.2019.7.42671 (PMC6754196; doi:10.5811/westjem.2019.7.42671)
Supplement: Supplementary file 1 [file wjem-20-740-s001.docx]

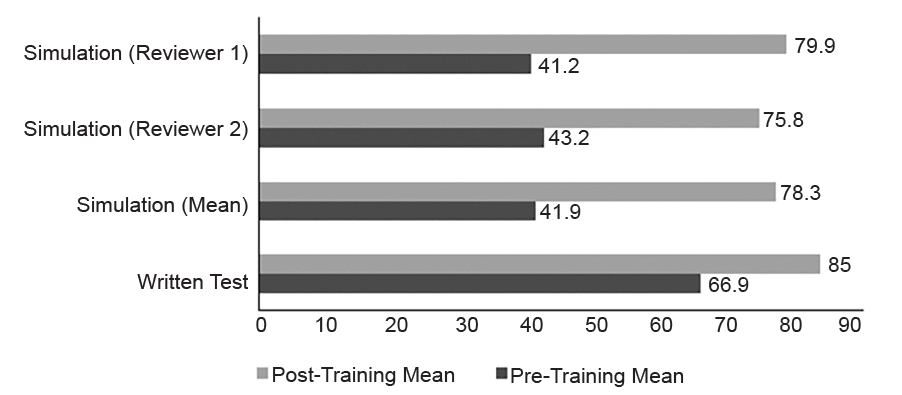
**Appendix 1**

**PTSD Checklist (PCL-C)**

Please read each one carefully, then **circle** one of the numbers to the right to indicate how much you have been **bothered** by the problem **in the past month.**

| Set B | Not at all | A little bit | Moderately | Quite a bit | Extremely |
| --- | --- | --- | --- | --- | --- |
| 1. Repeated disturbing memories, thoughts, or images of the stressful experience? | 1 | 2 | 3 | 4 | 5 |
| 2. Repeated, disturbing dreams of the stressful experience? | 1 | 2 | 3 | 4 | 5 |
| 3. Suddenly acting or feeling as if the stressful experience were happening again (as if you were reliving it)? | 1 | 2 | 3 | 4 | 5 |
| 4. Feeling very upset when something reminded you of the stressful experience? | 1 | 2 | 3 | 4 | 5 |
| 5. Having physical reactions (e.g., heart pounding, trouble breathing, or sweating) when something reminded you of the stressful experience? | 1 | 2 | 3 | 4 | 5 |
| **Set C** |  |  |  |  |  |
| 6. Avoiding thinking about or talking about the stressful experience or avoiding having feelings related to it? | 1 | 2 | 3 | 4 | 5 |
| 7. Avoiding activities or situations because they remind you of the stressful experience? | 1 | 2 | 3 | 4 | 5 |
| 8. Trouble remembering important parts of the stressful experience? | 1 | 2 | 3 | 4 | 5 |
| 9. Loss of interest in activities that you used to enjoy? | 1 | 2 | 3 | 4 | 5 |
| 10. Feeling distant or cut off from other people? | 1 | 2 | 3 | 4 | 5 |
| 11. Feeling emotionally numb or being unable to have loving feelings for those close to you? | 1 | 2 | 3 | 4 | 5 |
| 12. Feeling as if your future will somehow be cut short? | 1 | 2 | 3 | 4 | 5 |
| **Set D** |  |  |  |  |  |
| 13. Trouble falling or staying asleep? | 1 | 2 | 3 | 4 | 5 |
| 14. Feeling irritable or having angry outbursts? | 1 | 2 | 3 | 4 | 5 |
| 15. Having difficulty concentrating? | 1 | 2 | 3 | 4 | 5 |
| 16. Being “super alert” or watchful or on guard? | 1 | 2 | 3 | 4 | 5 |
| 17. Feeling jumpy or easily startled? | 1 | 2 | 3 | 4 | 5 |

Add up all items from each of the 17 items for a total severity score (range = 17-85)

*****PTSD is defined using DSM criteria for a diagnosis: Symptomatic response to at least 1 “B” item, at least 3 “C” items, and at least 2 “D” items. Symptomatic responses are the categories of Moderately or above.

17-29 This cut off shows little to no severity.

28-29 Mild PTSD symptoms.

30–44 Moderate to Moderately High severity of PTSD symptoms.

45-85 High Severity of PTSD symptoms.
