# Supplementary material for: Prevalence of Post-Traumatic Stress Disorder in Emergency Physicians in the United States
Source: West J Emerg Med. 2019 Aug 28;20(5):740–6. doi: 10.5811/westjem.2019.7.42671 (PMC6754196; doi:10.5811/westjem.2019.7.42671)
Supplement: Supplementary file 2 [file wjem-20-740-s002.docx]

**Appendix 2: PTSD among Emergency Department physicians (N = 526)**

| **PTSD Component** | **n (%)** |
| --- | --- |
| Set B |  |
| Repeated disturbing memories, thoughts, images of stressful experience  Not at all  A little  Moderately  Quite a bit  Extremely | 152 (28.9)  228 (43.3)  99 (18.8)  37 (7.0)  10 (1.9) |
| Repeated disturbing dreams of stressful experience  Not at all  A little  Moderately  Quite a bit  Extremely | 295 (56.1)  151 (28.7)  54 (10.3)  18 (3.4)  8 (1.5) |
| Suddenly acting or feeling as if the stressful experience were happening again  Not at all  A little  Moderately  Quite a bit  Extremely | 349 (66.3)  123 (23.4)  40 (7.6)  13 (2.5)  1 (0.2) |
| Feeling very upset when something reminded you of stressful experience  Not at all  A little  Moderately  Quite a bit  Extremely | 187 (35.6)  212 (40.3)  80 (15.2)  35 (6.7)  12 (2.3) |
| Having physical reactions (e.g. heart pounding, trouble breathing, or sweating) when something reminded you of the stressful experience  Not at all  A little  Moderately  Quite a bit  Extremely | 309 (58.7)  139 (26.4)  50 (9.5)  23 (4.4)  5 (1.0) |
| Set C |  |
| Avoiding thinking about or talking about the stressful experience or avoiding having feelings related to it  Not at all  A little  Moderately  Quite a bit  Extremely | 242 (46.0)  151 (28.7)  78 (14.8)  41 (7.8)  14 (2.7) |
| Avoiding activities or situations because they remind me of stressful experience  Not at all  A little  Moderately  Quite a bit  Extremely | 336 (63.9)  118 (22.4)  41 (7.8)  23 (4.4)  7 (1.3) |
| Trouble remembering important parts of the stressful experience  Not at all  A little  Moderately  Quite a bit  Extremely | 402 (76.4)  79 (15.0)  34 (6.5)  10 (1.9)  1 (0.2) |
| Loss of interest in activities that you used to enjoy  Not at all  A little  Moderately  Quite a bit  Extremely | 275 (52.4)  135 (25.7)  76 (14.4)  25 (4.8)  14 (2.7) |
| Feeling distant or cut off from other people  Not at all  A little  Moderately  Quite a bit  Extremely | 218 (41.4)  159 (30.2)  76 (14.4)  50 (9.5)  23 (4.4) |
| Feeling emotionally numb or being unable to have loving feelings for those close to you  Not at all  A little  Moderately  Quite a bit  Extremely | 273 (52.1)  133 (25.4)  64 (12.2)  37 (7.1)  17 (3.2) |
| Feeling as if your future will somehow be cut short  Not at all  A little  Moderately  Quite a bit  Extremely | 264 (50.2)  118 (22.4)  68 (12.9)  52 (9.9)  24 (4.6) |
| Set D |  |
| Trouble falling or staying asleep  Not at all  A little  Moderately  Quite a bit  Extremely | 160 (30.5)  166 (31.6)  100 (19.0)  62 (11.8)  37 (7.0) |
| Feeling irritable or having angry outbursts  Not at all  A little  Moderately  Quite a bit  Extremely | 158 (30.0)  209 (39.7)  94 (17.9)  47 (8.9)  18 (3.4) |
| Having difficulty concentrating  Not at all  A little  Moderately  Quite a bit  Extremely | 216 (41.1)  203 (38.6)  66 (12.5)  31 (5.9)  10 (1.9) |
| Being “super alert” or watchful or on guard  Not at all  A little  Moderately  Quite a bit  Extremely | 276 (52.5)  137 (26.0)  54 (10.3)  45 (8.6)  14 (2.7) |
| Feeling jumpy or easily startled  Not at all  A little  Moderately  Quite a bit  Extremely | 345 (65.6)  109 (20.7)  33 (6.3)  35 (6.7)  4 (0.8) |
| **PTSD Level** |  |
| PTSD Severity Score, mean (SD) | 31.1 (11.7) |
| PTSD*  Yes  No | 83 (15.8)  443 (84.2) |

*****PTSD is defined using DSM criteria for a diagnosis: Symptomatic response to at least 1 “B” item, at least 3 “C” items, and at least 2 “D” items. Symptomatic responses are the categories of Moderately or above.
