# Supplementary material for: Prevalence of Post-Traumatic Stress Disorder in Emergency Physicians in the United States
Source: West J Emerg Med. 2019 Aug 28;20(5):740–6. doi: 10.5811/westjem.2019.7.42671 (PMC6754196; doi:10.5811/westjem.2019.7.42671)
Supplement: Supplementary file 3 [file wjem-20-740-s003.docx]

| Appendix 3  Relationship between PTSD severity score* and predictor variables | | | | |  | |
| --- | --- | --- | --- | --- | --- | --- |
|  | Bivariate Models** | |  | Adjusted Model (R² = 0.043) | | |
|  | e^B(95% CI of e^B) | p-value |  | e^B(95% CI of e^B) | p-value | |
| **Demographic Factors** | |  |  |  |  | |
| Age (years) |  |  |  |  |  | |
| 22-35 | reference |  |  | reference |  | |
| 36-49 | 1.06(0.98 - 1.15) | 0.145 |  | 1.05 (0.97 - 1.14) | 0.213 | |
| >50 | 1.05(0.97 - 1.14) | 0.257 |  | 1.02 (0.93 - 1.1) | 0.737 | |
| Marital Status |  |  |  |  |  | |
| Married/Partner | 0.93(0.85 - 1.01) | 0.082 |  | 0.92 (0.85 - 1) | 0.052 | |
| Single | reference |  |  | reference |  | |
| Military |  |  |  |  |  | |
| Yes | 1.09(0.99 - 1.19) | 0.068 |  | 1.05 (0.96 - 1.15) | 0.251 | |
| No | reference |  |  | reference |  | |
| Victim |  |  |  |  |  | |
| Yes | 1.18(1.09 - 1.28) | <0.001 |  | 1.16 (1.07 - 1.26) | <0.001 | |
| No | reference |  |  | reference |  | |
| **Work Place Factors** |  |  |  |  |  | |
| Trauma level |  |  |  |  |  | |
| None | reference |  |  | reference |  | |
| Level 1 | 1.02(0.94 - 1.11) | 0.593 |  | 1.07 (0.97 - 1.17) | 0.185 | |
| Level 2 | 1.09(1.01 - 1.19) | 0.037 |  | 1.1 (1.01 - 1.2) | 0.026 | |
| Level 3/4 | 1.1(1.01 - 1.2) | 0.031 |  | 1.08 (0.99 - 1.18) | 0.1 | |
| Location of work |  |  |  |  |  | |
| Urban | reference |  |  | reference |  | |
| Suburban | 1.01(0.95 - 1.08) | 0.0682 |  | 1.03 (0.96 - 1.11) | 0.4 | |
| Rural | 1.08(0.98 - 1.2) | 0.106 |  | 1.09 (0.98 - 1.22) | 0.102 | |
| Board Certified EM | |  |  |  |  | |
| Yes | 1.09(0.97 - 1.22) | 0.162 |  | 1.02 (0.9 - 1.15) | 0.763 | |
| No | reference |  |  | reference |  | |
| Board Peds |  |  |  |  |  | |
| Yes | 0.81(0.67 - 0.97) | 0.023 |  | 0.83 (0.68 - 1.01) | 0.065 | |
| No | reference |  |  | reference |  | |
| *In terms of the ln(PTSD score). Thus coefficients reported are e^B.  **Non-significant (p > 0.20) bivariate covariates included: Sex, Children, Years of service, Board certified in Family medicine, Board certified in Internal medicine, Board certified in Pediatric medicine | | | | | |  |
