# Supplementary material for: Prevalence of Post-Traumatic Stress Disorder in Emergency Physicians in the United States
Source: West J Emerg Med. 2019 Aug 28;20(5):740–6. doi: 10.5811/westjem.2019.7.42671 (PMC6754196; doi:10.5811/westjem.2019.7.42671)
Supplement: Supplementary file 4 [file wjem-20-740-s004.docx]

| Appendix 4  Relationship between PTSD diagnosis and predictor variables | | | | |  |
| --- | --- | --- | --- | --- | --- |
|  | Bivariate Models* | |  | Adjusted Model (Nagelkerke R² = 0.078) | |
|  | OR (95% CI of OR) | p-value |  | OR (95% CI of OR) | p-value |
| **Demographic Factors** | |  |  |  |  |
| Age (years) |  |  |  |  |  |
| 22-35 | reference |  |  | reference |  |
| 36-49 | 1.54 (0.75 - 3.16) | .241 |  | 1.07 (0.41 - 2.891) | 0.892 |
| >50 | 2.68 (1.3 - 5.49) | .007 |  | 1.42 (0.41 - 4.94) | 0.582 |
| Sex |  |  |  |  |  |
| Male | 1.29 (0.8 - 2.09) | 0.294 |  | 1.05 (0.61 - 1.79) | 0.873 |
| Female | reference |  |  | reference |  |
| Marital Status |  |  |  |  |  |
| Married/Partner | 0.68 (0.37 - 1.25) | .216 |  | 0.64 (0.34 - 1.22) | 0.178 |
| Single | reference |  |  | reference |  |
| Military |  |  |  |  |  |
| Yes | 2.07 (1.13 - 3.81) | .019 |  | 1.54 (0.79 - 3.00) | 0.202 |
| No | reference |  |  | reference |  |
| Victim |  |  |  |  |  |
| Yes | 2.65 (1.53 - 4.58) | .000 |  | 2.16 (1.21 - 3.86) | 0.009 |
| No | reference |  |  | reference |  |
| **Work Place Factors** |  |  |  |  |  |
| Years of Service | |  |  |  |  |
| 0-5 | reference |  |  | reference |  |
| 6-11 | 1.34 (0.61 - 2.95) | .473 |  | 1.14 (0.44 - 2.98) | 0.789 |
| 12-17 | 2.3 (1.02 - 5.19) | .046 |  | 1.60 (0.54 - 4.75) | 0.402 |
| 18-23 | 2.81 (1.2 - 6.54) | .017 |  | 1.72 (0.49 - 5.97) | 0.395 |
| > 23 | 2.64 (1.21 - 5.76) | .015 |  | 1.53 (0.41 - 5.73) | 0.529 |
| Board Certified EM | |  |  |  |  |
| Yes | 2.29 (0.69 - 7.62) | .178 |  | 1.82 (0.52 - 6.28) | 0.347 |
| No | reference |  |  | reference |  |

*Non-significant (p > 0.20) bivariate covariates included: Children, Trauma level, location of work, Board certified in Family medicine, Board certified in Internal medicine, Board certified in Pediatric medicine
